# Aligning rhetoric with reality: a qualitative analysis of multistakeholder initiatives in the global food system

**DOI:** 10.1093/heapro/daae165

**Published:** 2024-12-19

**Authors:** Amber van den Akker, Anna B Gilmore, Alice Fabbri, Cecile Knai, Harry Rutter

**Affiliations:** Department for Health, University of Bath, Claverton Down, Bath BA2 7AY, UK; Department for Health, University of Bath, Claverton Down, Bath BA2 7AY, UK; Department for Health, University of Bath, Claverton Down, Bath BA2 7AY, UK; Faculty of Public Health and Policy, London School of Hygiene & Tropical Medicine, Keppel St, London WC1E 7HT, UK; Department of Social & Policy Sciences, University of Bath, Claverton Down, Bath BA2 7AY, UK

**Keywords:** multistakeholderism, commercial determinants of health, food system, global governance

## Abstract

Global food system governance increasingly relies on multistakeholder initiatives (MSIs) that aim to include those who are affected by and/or affected by an issue. Multistakeholderism’s perceived legitimacy is premised on both its outcomes (output legitimacy) and processes (input legitimacy), the latter in turn based on four key rationales: inclusiveness, procedural fairness, consensual orientation and transparency. To date, evidence on the ineffectiveness of MSI’s outcomes undermines its claims to output legitimacy. While individual case study assessments have also raised concerns over their processes, documenting instances of power asymmetries and corporate capture, there has hitherto been no comprehensive assessment of the input legitimacy of multistakeholderism. This work addresses that gap through interviews with 31 participants working either in or on MSIs. Participants noted significant challenges related to input legitimacy, including that (i) inclusion was often based on pre-existing networks of an MSI’s founders—most of whom were based in the global North—and risked excluding less well-resourced or marginalized actors; (ii) pre-existing power imbalances, both internal and external to the MSI, considerably influenced its processes and structures; (iii) goal-setting was complicated by conflicts of interest and (iv) reliance on informal processes limited transparency. The similarities in challenges across MSIs indicate that these are not attributable to shortcomings of individual MSIs but are instead indicative of wider system constraints. Rather than rely on multistakeholderism as a ‘good’ governance norm, our findings add to evidence that MSIs do not meet output legitimacy and signal that the legitimacy of MSIs in their current form should be questioned.

**Contribution to Health Promotion**

- Global food governance increasingly relies on multistakeholder initiatives (MSIs) despite concerns over their effectiveness and the quality of their processes. While evidence indicates ineffective outcomes (output legitimacy) of MSIs, a more comprehensive evaluation of their processes (input legitimacy) is needed.
- This study used qualitative analysis of interview data with 31 participants to examine four key rationales underpinning this input legitimacy: inclusiveness, procedural fairness, consensual orientation and transparency.
- We found that participants across MSIs consistently experienced significant challenges related to these four rationales. MSIs struggled to be inclusive, faced power asymmetries and lacked clear accountability structures, raising significant further doubts about their legitimacy.

## BACKGROUND

It is well established that the challenges driving the current unsustainable, unhealthy and inequitable global food system are not solvable through technical fixes but require a more fundamental political and economic ‘transformation’ of the global food system, including a shift in the norms, power relations and structures in its governance (Conti *et al*., 2021; Leeuwis *et al*., 2021; Béné, 2022). One response to the complexity of achieving such a transformation is the clear yet contested shift towards multistakeholder approaches within global food governance (McKeon, 2017; Chandrasekaran *et al*., 2021). While there remains a level of ambiguity around the term ‘multistakeholderism’, we use the definition proposed by the High-Level Panel of Experts on Food Security and Nutrition (HLPE) of the Committee on World Food Security—‘any collaborative arrangement between stakeholders from two or more different spheres of society (public sector, private sector and/or civil society), pooling their resources together, sharing risks and responsibilities in order to solve a common issue, to handle a conflict, to elaborate a shared vision, to realize a common objective, to manage a common resource and/or to ensure the protection, production or delivery of an outcome of collective, and/or, public interest’ (HLPE, 2018). Multistakeholder initiatives (MSIs) have been used for a variety of purposes, from shaping global policy solutions through the United Nations (UN) Food Systems Summit to setting business standards through the Forest Stewardship Council and governing corporate social responsibility through the UN Global Compact (Fransen and Kolk, 2007; Berliner and Prakash, 2012; Moog *et al*., 2015). Reflecting its increasing popularity, multistakeholder governance has been described as a ‘new blueprint of transnational coordination’ (Hofmann, 2020), with multistakeholder partnerships enshrined in the UN Sustainable Development Goal (SDG) 17 as a goal in itself (United Nations, 2015; UNDESA & The Partnering Initiative, 2020). Fundamentally, the institutionalization of multistakeholderism represents a normative shift in how global public problems *ought* to be addressed and by whom, with implications for which actors, problem framings and solutions are perceived as legitimate contributors to global food system governance (Ralston *et al*., 2023; Taggart and Abraham, 2024).

In order to maintain credibility and support for their role and the solutions they propose, non-state institutions such as MSIs have to actively establish and maintain their legitimacy, defined here as the perception that a governing organization has a right to rule and exercises it appropriately (Suchman, 1995; Bernstein, 2011). Building on prior literature on the legitimacy of multistakeholder governance, we distinguish between two sources of legitimacy: the quality of its processes (*input* legitimacy) and the quality of its outcomes (*output* legitimacy) (Scharpf, 1997; Bernstein and Cashore, 2007). Case study evidence on the outcomes of national and global multistakeholder approaches has repeatedly shown that these have resulted in ineffective, unambitious solutions, often relying on voluntary self-regulation, that were inconsistently adhered to (Berliner and Prakash, 2014; Knai *et al*., 2015; Rosewarne *et al*., 2020). A recent review of global multistakeholder platforms seeking to work towards the SDGs found alarmingly low levels of activity and a general lack of understanding of how partnerships may lead to transformational change (Widerberg *et al.*, 2023).

Despite evidence on the ineffective outcomes of multistakeholder approaches, multistakeholderism remains increasingly adopted as a norm of ‘good governance’ and sees a growing prominence in global governance (WHO, 2014; Hofmann, 2020; UNDESA & The Partnering Initiative, 2020). To account for this, many point out that the legitimacy of governance institutions is to a large extent driven by the perceived appropriateness of its processes and rules in relation to wider norms around what constitutes ‘good’ governance (Bernstein and Cashore, 2007; Bernstein, 2011). The legitimacy of multistakeholder processes is often justified through four key rationales, summarized by Mena and Palazzo (2012) as: (i) *Inclusion*—whether all relevant stakeholders are included and whether the involved stakeholders are representative of the issues at stake; (ii) *Procedural fairness*—relating to the neutralization of power differences in decision-making; (iii) *Consensual orientation*—is there a culture of cooperation, mutual agreement and reasonable disagreement? And (iv) *Transparency*—transparency of structures, processes and results of the MSI (Table 1) (Mena and Palazzo, 2012, p. 539).

**Table 1:** Criteria of multistakeholder initiatives’ democratic legitimacy, adapted from Mena and Palazzo (2012)

Dimension	Criterion	Definition
Input legitimacy	Inclusion	Involvement of stakeholders affected by the issue in the structures and processes of the MSI
	Procedural fairness	Neutralization of power differences in decision-making structures
	Consensual orientation	Culture of cooperation and reasonable disagreement
	Transparency	Transparency of structures, processes and results
Output legitimacy	Coverage	Number of rule-targets following the rules
	Efficacy	Fit of the rules to the issue
	Enforcement	Practical implementation of the rules and their verification procedures

Although these rationales are often used as justifications for multistakeholder approaches (Dentoni *et al*., 2018; Kalibata, 2020), there is a lack of empirical research analysing them. Aside from some notable exceptions related specifically to multistakeholder internet governance (Hofmann, 2016) and democracy (Gleckman, 2018), there remains a lack of critical research examining the extent to which these claims to legitimacy are borne out in practice (Nonet *et al*., 2022). Doing so is particularly relevant in the context of global food system governance given the importance of including different stakeholders and addressing power imbalances between these stakeholders for the legitimacy and foreseen effectiveness of global food system change (Conti *et al*., 2021; Montenegro De Wit *et al*., 2021). To address this gap in the literature, this study draws on interviews with participants working in and on MSIs in the context of the global food system with the aim to assess the rationales underpinning the legitimacy of MSIs’ processes and identify potential challenges related to the legitimacy of multistakeholder governance.

## METHODS

### Sample recruitment

We identified potential participants through the websites of global MSIs that aim to create healthier and more sustainable food systems and both peer-reviewed and grey literature written on relevant MSIs. During interviews, some participants recommended additional interview participants, whom we subsequently invited. Through our purposive sampling, we aimed to include a set of participants with diverse professional backgrounds and experiences to provide relevant perspectives on MSIs. The researcher conducting the interviews had no prior relationship with any of the participants. We limited our scope to MSIs in the context of the global food system to ensure a shared context between participants. We purposively selected participants based on their work in, with or relating to relevant MSIs and sought to include participants working in different sectors (e.g. academia, private sector), as well as participants who were employed full-time in the MSI, often funded through the organizations hosting or funding the MSI, including international organizations. While some MSIs included in our sample were founded relatively recently, others have operated for years. Participants worked at all levels of the MSIs and were based across the globe. Participants were asked to sign an informed consent form prior to participation and declare any financial conflicts of interest with the tobacco, alcohol, gambling or ultra-processed food industries for 5 years prior to the interview. Having such conflicts of interest did not exclude participants from this study. Ethical approval for this study was granted by the Research Ethics Approval Committee for Health (REACH) at the University of Bath (EP22/034).

### Data collection

All interviews took place remotely using Microsoft Teams and were recorded with participants’ permission. We developed an interview guide informed by the existing peer-reviewed literature, notably Mena and Palazzo’s (2012) framework on assessing democratic legitimacy in the context of MSIs (Table 1) because of its concise yet comprehensive encapsulation of many of the legitimacy criteria of MSIs described in the literature (Mena and Palazzo, 2012). The interview guide explored participants’ experiences with MSIs, their reflections on the supposed risks and benefits of multistakeholder governance as identified in the peer-reviewed literature and views on the role and responsibilities of multistakeholder governance within the wider global food system governance context. The interview guide was developed by the lead author (A.v.d.A.) and iteratively revised through various meetings with the research team as well as following pilot interviews with two participants who met the inclusion criteria but were not subsequently included in this study. The interview guide is available in Supplementary File S1.

### Data analysis

We used critical thematic analysis, shifting between inductive and deductive modes, to explore participants’ reflections on—and challenges related to—the legitimacy of MSIs (Braun and Clarke, 2022). For the deductive aspect of the analysis, we used the framework by Mena and Palazzo (2012) to guide the development of subthemes based on the experiences and challenges that participants described. In addition, we used inductive thematic analysis to develop themes from participants’ reflections, drawing on our interview data to identify areas of focus for participants that were not captured in the framework described above (Table 1). Through an iterative process of coding the interview transcripts and discussing potential themes with the research themes, we developed themes that focused on the role of multistakeholder approaches in global food governance more broadly. All themes and subthemes that were developed during analysis are available in Supplementary File S2 and are described in the *Results* section.

This qualitative research was grounded in constructivism, as we understand the legitimacy of multistakeholder governance to be fundamentally constructed, interpreted and re-interpreted in a social system where ideas, norms and discourse have significant influence (Finnemore and Sikkink, 2001). Using this constructivist lens, we aimed not to establish a truth about the legitimacy of MSIs, but rather to explore the multiple subjectively constructed realities that participants report. Our critical approach to this topic was shaped by the extensive literature on the need for global food governance to fundamentally address power, including the potential problematic role of commercial actors, in order to be able to create a sustainable, healthy and just global food system (McKeon, 2017; Swinburn *et al*., 2019; Baker *et al*., 2021; Clapp, 2021; Baudish *et al*., 2024).

All interviews were conducted in English and transcribed by the lead author (A.v.d.A.). NVivo V.12 software was used for data management and coding. A.v.d.A. conducted the initial coding and four members of the research team (H.R., A.F., C.K., M.K.) independently coded a subset of transcripts. A.v.d.A. coded all transcripts using the approach outlined above, and all authors reviewed memos including illustrative quotes for each theme, discussing these iteratively to reach consensus across the research team.

## RESULTS

### Participant characteristics

We emailed 56 interview candidates, of whom 19 did not respond, six declined to participate due to time constraints and 31 agreed to be interviewed. Interviews took place between October 2022 and March 2023 and lasted on average 60 min. Participant characteristics are reported in Table 2. Participants jointly had experiences with over 18 different MSIs, all of which were global in scope. Seven participants reported that they have or have had financial or professional ties with the tobacco, alcohol, food/beverage or gambling industry in the past 5 years. There was no clear relationship between the sector a participant worked in and their views on multistakeholderism. We did note that participants who worked in MSIs at the time of the interview were at times more hesitant to express negative opinions about the multistakeholder process and stressed the need for anonymity before doing so. Most participants discussed the MSI they worked with, although some expressed views on the wider system within which multistakeholder approaches take place.

**Table 2:** Participant characteristics

Characteristics	No. of interviewees (%)
Working in MSI	
Yes	21 (68)
No	10 (32)
Type of primary institution	
Multistakeholder initiative	9 (29)
NGO	9 (29)
University	6 (19)
International organization	3 (10)
Investor	2 (6)
Transnational food industry	2 (6)
Location of primary institution	
Europe	18 (58)
North America	8 (26)
South America	0 (0)
Africa	3 (10)
Asia	1 (3)
Oceania	1 (3)
Financial conflicts of interest of the interviewee	
Yes	7 (23)
No	24 (77)

### Participant experiences related to the criteria for input legitimacy

#### Inclusion

*The face-to-face meeting, immediately [LMIC stakeholder] has already said sorry, we can’t afford to travel. And nobody’s going to pick up that bill for them to travel. So we’ve immediately lost one of our stakeholder groups, unless we make an effort to find a way of engaging them somehow, just by wanting to have a face to face meeting. (MSI)*

Although many of the included MSIs listed the organizations and/or individuals involved in the MSI on their website, there was a lack of transparency on how these stakeholders were chosen and many MSIs did not have specific inclusion and exclusion criteria for which actors were able to join the MSI. During the interviews, many participants reiterated the rationale for inclusion, i.e. that all relevant actors ought to be included, as a key reason to engage in multistakeholder approaches. However, when reflecting on their experiences, nearly all participants related the difficulty of building a truly inclusive multistakeholder process. Participants noted significant capacity and resource barriers that many actors, particularly those at the local level and from the global South, face when participating in global MSIs that are often initiated by and located in high-income countries. Participants reflected that those initiating an MSI would often invite actors that were already in their networks, which often led to the recruitment of well-connected, powerful actors. Although some MSIs sought to include actors from the global South, participants noted that such actors that were ultimately included were those who already had ties with the actors initiating the MSI and were less likely to adopt a critical stance towards the multistakeholder process, particularly if their pre-existing ties included financial dependence. Many participants outside MSIs noted that MSIs were not truly inclusive of those most important actors and groups while participants inside MSIs similarly questioned whether they had the right actors around the table. Yet many participants expressed the need for a trade-off to be made between inclusion and moving forward, and not all saw the lack of inclusivity as a particular limitation.

#### Procedural fairness

*Saying everyone’s concerns and everyone’s needs [are discussed] within one space, one table, fails to recognise that workers […] do not have the power to sit at the table equitably. (NGO)*

Procedural fairness reflects the extent to which power relations between participants are neutralized so that all included stakeholders are equally able to influence the multistakeholder process (Mena and Palazzo, 2012). While some participants discussed attempting to address existing power asymmetries through careful facilitation of discussions, many considered it impossible to achieve true equality in multistakeholder processes when the wider global food system context suffers from significant power imbalances, exploitation and distrust between different actors and sectors. As examples of power asymmetries, participants mentioned the language used, which some actors may struggle to understand, and dependent relationships outside of the initiative. For example, farm workers sitting alongside representatives from the corporation that buys their products may limit the workers’ ability to safely and freely discuss a topic. Some participants moreover noted that high levels of distrust between different sectors in the global food system made equal and open participation of all actors exceedingly difficult to facilitate.

One often-mentioned power imbalance was financial, as participants noted that financial structures outside the multistakeholder process, including who is able to give and receive funding and what requirements there are to obtain funding, inevitably impacted the multistakeholder structure and process. Many noted how the current context of constrained funding allows those with financial power to shape the multistakeholder process and its outcomes to their benefit, while those dependent on this funding may be hesitant to speak freely or leave an initiative if they do not agree with its direction, as this may mean a significant loss of resources. Not all MSIs included in the study sample involved commercial actors, which was in some cases a conscious decision due to the risk of their being perceived to have undue influence on the multistakeholder process. Of those who did involve commercial actors, some participants reflected that even though they might have hesitations regarding this engagement, they saw this as inevitable due to the concentrated power and resources within the private sector. A few participants moreover expressed concerns that if they did not leverage these resources within their initiative, they would risk having these resources leveraged against them.

#### Consensual orientation

*The quote I like is “we shouldn’t let perfect be the enemy of good”. And so to me that is where it is. How do we find middle ground? How do we find things that are relatively acceptable and palatable to all sides? Not perfect. Maybe not even great. But [that] at least get us moving. (Business-led MSI)*

The ability of MSIs to reach a consensus among participants, or at least reach a position of cooperation despite dissenting positions (Mena and Palazzo, 2012), was used by a number of participants to justify the use of multistakeholderism. Almost all participants considered the absence of strategic alignment to be detrimental to the multistakeholder process. However, participants noted that it is exceedingly difficult to reach such alignment between all partners in a multistakeholder process, as partners might fundamentally disagree on the goals for the initiative. Some participants related that they overcame these difficulties by seeking compromises on these goals, even though they might as a result become less ambitious. Some participants perceived this dilution of the MSIs’ goals to be a fundamental problem, highlighting the risk that multistakeholder working may at times hold up progress by going for solutions that may be relatively palatable for all actors but that do not go far enough to address the root causes of the challenges facing the global food system, particularly when these require a shift in power. Moreover, a few participants noted that only including one or a few ‘stakeholders’ from a sector in the MSI does not adequately reflect the range and variety of goals held by different actors in this sector. This concern was particularly raised in relation to the inclusion of civil society and affected community stakeholders, which previous research indicates are often least represented in global food MSIs (Slater *et al*., 2024; Van Den Akker *et al*., 2024). Some participants expressed doubts as to whether the goals of the MSI were aligned with the priorities of those affected by the issue, especially when there was a lack of inclusion of affected communities and critical voices.

In addition, participants expressed concerns that the ability of MSIs to have a consensual process was severely limited by conflicts of interest of some partners in the MSI. While such conflicts of interest can occur in all sectors, the potential for conflicts of interest was considered to be particularly pronounced when engaging commercial actors who derive their profit from unhealthy and unsustainable products. Some participants, predominantly those who worked in MSIs that included commercial actors, emphasized that MSIs have the potential to create ‘win-win’ scenarios for both public and private sectors. However, most participants saw such conflicts of interest as a significant risk in MSIs, often linking this to reputational damage or the aforementioned dilution of goals. Some questioned the motivation for commercial actors to join MSIs, suspecting some commercial actors to participate in MSIs for reputation laundering purposes or to further delay action. Nevertheless, many participants still felt unable to exclude the private sector from MSIs, due to its power and resources, which presented a difficult trade-off. Instead, the potential for conflicts of interest led some actors to choose not to engage in MSIs themselves as, for them, the potential reputational risks of doing so outweighed the potential gains.

#### Transparency

*There is no transparency. The transparency is ad hoc. […] Where is the money coming from? Where is it going to? In particular what are the monetary and non-monetary transactions between major players and everybody else at the table? Things which we would not accept in the public domain. (Academic)*

Most MSIs discussed in this study did not have publicly available information about their specific outcomes or internal processes. In part, participants noted that this was a result of MSIs relying mostly on informal governance processes. Many MSIs did not have formal rules of engagement in place, nor did they require participants to report on their progress or contributions to the MSI. Instead, they relied on informal trust relationships rather than formal monitoring mechanisms, even though a few participants reflected on the risk of this approach, one participant noting that *‘it hasn’t gone wrong yet’* (MSI). This was in part due to the significant resources that would be required to conduct formal monitoring of the various actors that are included in an MSI, who have different levels of resources and capacity to dedicate to reporting. Some participants also remarked that they did not see monitoring as the role of the MSI. Furthermore, participants reflected on the added difficulty that, while quantitative data might be easier to monitor, this does not always reflect the type of outcomes that are relevant to the goals MSIs seek to address, particularly when seeking solutions to issues in the global food system, which might be more social or political in nature.

Despite this reliance on trust in many MSIs, participants noted at times a lack of trust between actors, often due to pre-existing tensions or past harmful interactions. Participants also noted that having transparency about the results of the MSI was made more difficult by the fact that they were not sure how to meaningfully show accountability in multistakeholder processes. In particular, the lack of inclusion of those who might meaningfully hold the multistakeholder process to account made it more difficult for MSIs to be transparent about their results and governance processes to those who would benefit from their actions. In addition, many participants indicated that they were not sure to whom a multistakeholder process *should* be accountable, given how stakeholders in the MSI were often accountable to entirely different constituencies. This led to questions about whom MSIs should be transparent about their governance processes and results.

### Reflections on the role of multistakeholder approaches in global food governance

We’re trying. *And I think there’s a difference between ambition and the goal, and the reality. And, you know, you hold the ambition, but you also hold some reality. And so I don’t want to seem naive and to think that if you just crack the mould you can now rebuild. Again, there’s a lot of incentives, disincentives on both sides, and complexity, to address. (MSI)*

While most participants recognized that there are significant discrepancies between the rationales used to justify the legitimacy of multistakeholder processes and their personal experiences with MSIs, many attributed this to shortcomings in their specific MSIs which they hoped to be able to overcome in the future. Most participants were not aware that these challenges were common across other MSIs. A few participants reflected on multistakeholder governance more generally, stating that they noticed a lack of consistent institutional support that would enable multistakeholder governance to work. Despite their concerns, many participants also expressed they saw ‘no other option’. Some participants saw multistakeholder governance as resulting from a failure of public institutions to remain free from corporate capture and hold society’s best interests at heart. Many participants saw the responsibility for representative and fair governance as solidly with states and international organizations and considered it problematic that these institutions have often failed to remain free from corporate interests. At the same time many participants indicated that, in their experience, MSIs are similarly constrained by existing incentives and disincentives which limit their ability to establish truly inclusive, fair, consensus-oriented and transparent governance structures and therefore significantly limit their ability to effectively drive systemic change. Recognizing the shortcomings in current implementations of multistakeholderism led some participants to write off multistakeholder working in general while others suggested that multistakeholderism may be a useful addition to other, potentially more confrontational, approaches that are not reliant on consensus among different sectors and actors with such vastly different power and interests.

Although many participants expressed a belief in multistakeholderism as a useful way forward for global food governance, some participants argued that multistakeholderism has broader detrimental consequences for global governance, warning in particular of the potential further entrenchment of power asymmetries and facilitation of corporate capture of global food system governance. Some participants thus reflected that, while they themselves participated in MSIs, they simultaneously recognized the risks associated with relying on multistakeholder approaches in light of the significant constraints this governance approach currently faces and were not sure whether multistakeholderism would be able to drive global food system transformation.

## DISCUSSION

This study is one of the first to empirically explore the rationales underpinning the legitimacy of multistakeholder governance processes and to do so across multiple MSIs. It found that participants across 18 MSIs consistently reported that four rationales underpinning the legitimacy of multistakeholder processes—inclusion, procedural fairness, consensual orientation and transparency—were not met. Participants reported similar challenges, although these led to different conclusions about the role of multistakeholderism in global food governance, with participants working in MSIs that collaborated with the private sector or who worked in the private sector themselves generally expressing a more optimistic view of multistakeholerism. Unlike previous case study work, our findings show that these challenges cannot be attributed to the shortcomings of individual MSIs but instead suggest that the legitimacy of multistakeholderism as ‘good’ governance is severely challenged.

The first rationale analysed in this study is the inclusion of all relevant stakeholders. Many participants noted difficulties with creating a truly inclusive multistakeholder process. These findings are supported by recent evidence on the networks of actors involved in two subsamples of global food MSIs, which found that these networks consist of mostly commercial and state actors, while civil society and marginalized communities remain on the fringes of this network (Slater *et al*., 2024; Van Den Akker *et al*., 2024). Similarly, prior case study research on MSIs has found that critical and minority voices were excluded from multistakeholder processes (Cheyns and Riisgaard, 2014; Clapp *et al*., 2021; Montenegro De Wit *et al*., 2021; Verhaeghe, 2023), either from necessity or, at times, by choice (EPHA, 2019; CSIPM, 2021). Research on multistakeholder platforms set up by the extractives industry has noted how these industries will use ‘inclusionary control’ to set up multistakeholder platforms that promise reform and influence in decision-making but where the terms of inclusion are set so that heterogeneous sectors are homogenized into one ‘stakeholder’ and dissenting voices within this sector are subdued or their proposals diluted (Verweijen and Dunlap, 2021; Verhaeghe, 2023). The normative implications of food system ‘transformation’ mean that differences in perspectives of what a ‘better’ food system looks like can fundamentally change the direction, extent and pace of food system change (Montenegro De Wit *et al*., 2021; Béné, 2022). Evidence shows that the extent to which different actors are able to input into governance has significant implications for the direction of change, including which issue framing gets taken up and which policy solutions are formulated (Campbell *et al*., 2020; Montenegro De Wit *et al*., 2021; Suzuki *et al*., 2021; Mesiranta *et al*., 2022). The findings from our study therefore highlight a potential risk related to which perspectives inform global food governance if it is driven by MSIs that lack inclusivity but who continue to derive legitimacy from the perception that they represent ‘inclusive’ governance (Bernauer and Gampfer, 2013; Verweijen and Dunlap, 2021).

The second rationale is that MSIs are able to provide a shared space free from existing power asymmetries. However, many participants noted that MSIs reflect rather than challenge existing power asymmetries. Examples included that the language used in discussions was not always accessible to all stakeholders, or the extent to which different stakeholders were able to take on a more or less strategic role in the MSI and safely express their concerns and criticisms in this role. These findings lend weight to prior research which found that civil society actors are less likely to be formally included than more powerful private-sector actors, and are often given roles in project implementation, advocacy and fundraising rather than as decision-makers or watchdogs (Storeng and De Bengy Puyvallée, 2018). Moreover, participants’ experiences highlighted the extent to which an MSIs’ goals and structures were inextricably linked to power asymmetries in the wider system. This included challenges due to existing relationships of distrust or because concentrated resources allowed some stakeholders to shape the structure and agenda of an MSI. The extent to which private-sector actors and their resources were considered essential to some MSIs’ ability to operate lends credence to concerns that multistakeholderism may institutionalize a reliance on private-sector actors as legitimate policy stakeholders and further embed corporate power in the global food system (Chandrasekaran *et al*., 2021; Lencucha, 2022; Ralston *et al*., 2023).

A third rationale underpinning the legitimacy of multistakeholder processes is that they lend themselves to discussions oriented towards consensus, or at least reasonable dissent, in service of shared goal-setting. Prior evaluations of partnerships emphasize the importance of having shared goals and a strong alignment of interest between partners as key enablers of success (Peters and Pierre, 2010; Gray and Purdy, 2018). However, many participants reflected on the difficulty of being consensus-orientated when actors have fundamentally different—at times opposing—interests. The impact of competing goals among actors, particularly related to private-sector actors who derive their profit from unhealthy and unsustainable products and services, has previously been flagged as a key obstacle to effective collaborative governance in the global food system (Kaan and Liese, 2011; Fanzo *et al*., 2021). In instances where the goals of participating organizations fundamentally conflict, many have argued that there is no basis for collaborative goal-setting (Donovan *et al*., 2015; McKeon, 2017). Indeed, our findings indicate a risk of MSIs’ goal being diluted due to the emphasis placed on consensus. This concern is further evidenced by prior research on a multistakeholder approach to obesity policy showing that this led to diluted policy goals (Lelieveldt, 2023). At the same time, avoiding private-sector collaboration altogether was not considered feasible nor desirable by many participants. The heterogeneity of the private sector means that some actors may be beneficial, if not essential, to global food system transformation and that it is overly simplistic to approach the ‘private sector’ as a whole as a barrier to change (Turnheim and Sovacool, 2020). Nevertheless, enabling food system transformation will require a shift in the significant power imbalance between these different sectors, which may be met with resistance from the actors whose base of power and resources is threatened (Turner *et al*., 2020; Clapp, 2021; Leeuwis *et al*., 2021).

The fourth rationale, that of transparency, was considered to be lacking by many participants external to an MSI. Participants reported that they were often unable to find reports of MSIs’ outcomes and processes as their reliance on informal governance processes meant that these were often not written down or publicly available. An added difficulty in reporting the outcomes of the MSIs was that many of the types of outcomes that are most relevant to global food system transformation are not easily measurable. Easily measurable targets such as product reformulation or nutrient enhancement do not address the fundamental problems facing the food system, which are driven by political priorities, power imbalances and social inequities that are more difficult to measure and report progress on (Van Bers *et al*., 2019; Ingram and Thornton, 2022). This was mentioned by various participants as a key challenge, as requirements from partners or funders to report on their outcomes created incentives for MSIs to focus on issues that allowed for monitoring and reporting, rather than focusing on truly addressing the fundamental issues facing the global food system.

The findings from this study align with prior research that has described the tensions between what multistakeholderism is ‘supposed to do’ and the realities of what its processes (input) and outcomes (output) are able to achieve (Hofmann, 2016; Gleckman, 2018). Given that existing evidence shows the ineffective outcomes from many multistakeholder approaches this article, by examining instead the processes of multiple MSIs, highlights that a multistakeholder approach is similarly unlikely to be able to establish and uphold legitimate governance processes. Instead, our findings indicate that multistakeholder approaches may risk further embedding existing problems, such as exclusivity, power imbalances and conflicts of interest. The fact that this study finds that MSIs do not adhere to the justifications for their legitimacy does not mean that these rationales and the narrative of why multistakeholder processes should work do not have a real impact (Hofmann, 2016). On the contrary, as Hofmann (2016) points out, such ‘political fictions’ have allowed multistakeholderism to become a goal in and of itself, with those working in multistakeholder governance arrangements placing a strong focus on procedures and narratives in order to construct legitimacy and protect the concept against ‘clashes with reality’ (Hofmann, 2016). Indeed, some have pointed out that adopting a multistakeholder approach implicitly lends legitimacy to a policy process (Lelieveldt, 2023; Ralston *et al*., 2023) which this study suggests is highly misleading.

The risks raised in this study become more pronounced in light of prior research, which has highlighted the high prevalence of powerful commercial actors in global food MSIs and has raised concerns about their ability to mitigate the risk of corporate capture (Chandrasekaran *et al*., 2021; Van Den Akker *et al*., 2024). In fact, the adoption of multistakeholderism as a governance norm in itself has been argued to be the result of powerful actors advocating for its use as a way to advance their interests (Taggart and Abraham, 2024). Research has repeatedly shown how corporations have promoted frames that include them as ‘part of the solution’ and pushed for multistakeholder approaches (Campbell *et al*., 2020; Lacy-Nichols and Williams, 2021; Mariath and Martins, 2022). Within a context of significant and growing concentration of, particularly corporate, power in the global food system, there is a risk that multistakeholder governance spaces further perpetuate these power asymmetries by enabling corporate capture of global governance while maintaining a veneer of equal access and input (Chandrasekaran *et al*., 2021; Clapp, 2021). While input legitimacy does not necessarily enable or negate corporate power, the inability of a governance process to be inclusive, fair and transparent may increase its susceptibility to corporate practices, power and potential capture (Madureira Lima and Galea, 2018; Ulucanlar *et al*., 2023).

This study has a number of limitations, one of which is that this study only focused on the rationales used to justify the legitimacy of MSIs’ governance processes, without including their outcomes as part of the analysis. Additionally, this study drew only on interview data, without significant triangulation with other types of data. While we tried to recruit participants evenly across sectors, some sectors—notably the private sector—were more difficult to recruit from. It would be a particularly useful area for future research to draw on a range of data to enable a more in-depth exploration of various MSIs’ specific governance structures, processes, contexts and outcomes, which was beyond the scope of this study. The current research raises a number of important questions, which future research might usefully seek to answer. One potential area for future research is to improve understanding of how the rationales for and norms around multistakeholderism have developed over time and across national and international contexts. A second important area for future work is the need for more guidelines on when and under what circumstances multistakeholder approaches may be appropriate and when they should be avoided, to provide guidance for those who are unsure whether to engage in MSIs.

## CONCLUSION

Multistakeholder approaches are often justified as providing inclusive and transparent governance processes, free from power imbalances, to facilitate shared goal-setting. Instead, our findings indicate that MSIs face significant challenges that limit their ability to establish and maintain processes in line with these rationales underpinning their legitimacy. MSIs’ processes are inevitably constrained by the wider governance systems in which they are embedded, including the conflicting interests and responsibilities, varying accountability structures and significant power imbalances that characterize these. By recording similar challenges across MSIs, the findings from this study indicate the importance of moving beyond the outcomes or processes of a specific MSI to instead consider the wider implications that adopting multistakeholderism as a governance norm poses for our ability to address the complex, fundamentally social, economic and political, problems facing the global food system. The trade-offs that MSIs often make, between requiring the resources of powerful actors to enable change while simultaneously wanting to be inclusive of traditionally marginalized perspectives and set ambitious goals to address the fundamental problems facing the global food system, point to a dilemma that is often at the core of global food system governance, not only multistakeholder governance. However, rather than rely on multistakeholderism as a ‘good’ governance norm, it is important to recognize these challenges and the potential risks these carry, particularly when the processes used in multistakeholder approaches are not considered legitimate. The challenges identified in this study therefore add significant weight to the evidence that multistakeholder approaches to global governance, as they are currently implemented, should be seriously questioned and revisited.

## Supplementary Material

daae165_suppl_Supplementary_Files_1

daae165_suppl_Supplementary_Files_2

## Data Availability

The data underlying this article cannot be shared publicly in order to protect the privacy of individuals participating in the study.
