# Supplementary material for: Aligning rhetoric with reality: a qualitative analysis of multistakeholder initiatives in the global food system
Source: Health Promot Int. 2024 Dec 19;39(6):daae165. doi: 10.1093/heapro/daae165 (PMC11655872; doi:10.1093/heapro/daae165)
Supplement: daae165_suppl_Supplementary_Files_1 [file daae165_suppl_supplementary_files_1.docx]

**Supplementary file 1: Interview topic guide**

*Notes: Not all questions were appropriate for all participants, and this guide was adapted as necessary.*

**Introduction**

Thank you again for agreeing to take part in an interview for our study today.

[interviewer introduce themselves and the study]

Are you happy that you understand the research project and what it will entail? Do you have any questions before we start? If not, I will now switch on the recording.

| **Introduction** | |
| --- | --- |
| Firstly, I’d like to know a little more about you and what it is that you do | |
| Could you tell me a bit about yourself, the initiative/organisation you work for and what your role is? | - What are the problem(s) that the initiative/organisation is set up to address? - What is the added benefit of working in a partnership to address this issue? |
| **Transparency** | |
| How do you measure the impact of your initiative? | - To whom do you report this impact and how? - Who else would you say the initiative is accountable to? - Who else would you say the initiative should be accountable to? |
| How important do you think it is that the initiative acts in the public interest and how do you ensure that it does? |  |
| **Inclusion** | |
| How do you make sure that all relevant stakeholders have a seat at the table? | - Who are the most relevant stakeholders? - Who is still missing at your current table, and why? |
| How do you choose which specific stakeholder is involved? | - Do you have any exclusion criteria for stakeholders wanting to join the initiative? |
| **Consensual orientation** | |
| In your experience, how important is it that all stakeholders have a shared goal? | - How did you achieve this, particularly when different stakeholders have different obligations to different constituencies? - How do you create a bridge when stakeholders may have different starting points to the same problem? - How do you guard against conflicts of interest? |
| Have you experienced a situation where there were different or conflicting perspectives or ideas? | - How were these resolved or managed? - How do you make sure that you have critical voices at the table as well? |
| **Procedural fairness** | |
| Have you experienced a situation where there were power imbalances between different stakeholders? | - If so, how did you manage these? - Was that sufficient? |
| Do you have principles of engagement in place for your members? | - If so, what do these contain? - If not, what would you say these should contain if you were to put these in place? |
| Do you have mechanisms in place to hold your members to account? |  |
| **General questions** | |
| What do you think potential risks of working in multistakeholder partnerships are? | - Are there any parts of food governance that you think multistakeholder working is particularly suited or not well-suited to? |
| How important is it that multistakeholder initiatives are embedded in wider global governance structures? | - How do your activities link to wider governance processes? - Are you collaborating with governments or international organisations and if so, how? |
| Aside from the structures and processes we talked about, what other aspects contribute to good practice in multistakeholder governance? |  |
| Why do you think multistakeholder approaches to global food system governance has become much more common in recent years? | - Why do you think this approach is preferred over, for example, a regulatory approach? |
| **Closing remarks** | |
| That’s all the questions I have for you today. Thank you again for your contribution. | |
| Is there anyone else you know that I should speak to about this? |  |
| Do you have any questions, or would you like to provide any further comments before I switch of the recorder? |  |
