# Supplementary material for: Aligning rhetoric with reality: a qualitative analysis of multistakeholder initiatives in the global food system
Source: Health Promot Int. 2024 Dec 19;39(6):daae165. doi: 10.1093/heapro/daae165 (PMC11655872; doi:10.1093/heapro/daae165)
Supplement: daae165_suppl_Supplementary_Files_2 [file daae165_suppl_supplementary_files_2.docx]

**Supplementary file 2.** Participants’ experiences with and reflections on multistakeholderism.

| **Participants' experiences with MSIs categorised according to the criteria for input legitimacy** | | |
| --- | --- | --- |
| **Criteria for input legitimacy (Mena and Palazzo, 2012)** | **Subthemes** | **Illustrative quotes** |
| **Inclusion**  Involvement of stakeholders affected by the issue in the structures and processes of the MSI | Multi-stakeholder initiatives are driven by, and mostly include, already powerful actors | “The caveat is that we have had good conversations with farmers, but I think, you know, it's not a large group of farmers. And those farmers were farmers that probably were working with the member companies” (Business-led MSI). |
|  | Barriers to inclusion for those who lack the resources, capacity or ability to join mostly Global North-led initiatives leads to a skewed member base | “The face-to-face meeting, immediately [LMIC stakeholder] has already said sorry, we can't afford to travel. And nobody's going to pick up that bill for them to travel. So we've immediately lost one of our stakeholder groups, unless we make an effort to find a way of engaging them somehow, just by wanting to have a face to face meeting” (MSI). |
|  | Referring to all partners in an MSI as ‘stakeholders’ as if they are equal is problematic in itself | “I think that by using the word stakeholder to describe constituencies, communities, affected peoples, citizens, loses, just on the choice of vocabulary, fundamental notions from which we have built what is called democracy today.” (Academic). |
|  | Multi-stakeholder initiatives do not include critical voices or more marginalised communities. | “Because the point of the multistakeholder initiative was to have like a really independent set of other stakeholders, in addition to affected communities, who are going to be able to speak out and call out companies when things are going wrong. And you're really just not seeing those actors at the table as much anymore” (NGO). |
| **Procedural fairness**  Neutralisation of power differences in decision-making structures | Existing power asymmetries outside multistakeholder initiatives are too great to have procedural fairness inside multistakeholder processes | “Basically what they are saying, everybody has a stake and everybody. But the problem with the stakes or the power is, like, everybody has a stick. But if your stick is 3 meters and the other one is 1 centimetre, how can you compete? You know? How can you be equal?” (Academic). |
|  | Existing power relations outside multistakeholder initiatives, particularly related to financial power, inevitably grant those with power more ability to steer the MSI. | “I think even when people have the best intentions or organisations have the best intentions, at the end of the day, that power dynamic is always present. And whoever holds the money inevitably has more power and can dictate the way projects can move” (Investor). |
|  | Power asymmetries already influence who is included in multistakeholder processes, out of fear for resistance from powerful actors | “I have my views on some large corporations which are mixed, just to put it diplomatically. But I also think that if you exclude them from change processes, they are going to fight tooth and nail to maintain the incumbent regime” (MSI).  “It's such a centralised place of money and power. Which makes it very difficult to steer and very tricky etcetera. But also really difficult. Yeah. I don't think you can ignore them, really” (NGO). |
|  | It is unrealistic to expect less powerful actors to engage with powerful actors as equals when there are clear power asymmetries outside the MSI, that may impact them beyond the multistakeholder process | “in practice, MSIs do not address the power imbalance of those different stakeholders. And thus, saying everyone's concerns and everyone's needs within one space, one table, fails to recognise that workers in […] do not have the power to sit at the table equitably and do not have, in MSIs as they exist, do not get the power to decide” (NGO). |
| **Consensual orientation**  Culture of cooperation and reasonable disagreement | It is difficult to achieve strategic alignment when partners have fundamentally different conceptualisations of the problem and ideas of what the goal should be. | “I think that the individual organizations around the table have multiple goals, some of which have an overlap, but it is not a statement of shared goals across the organisation. And I think that's important because you find tugs going on, and many of those tugs and battles within multi stakeholder groups are over fundamental issues of what is the role. What's the nature of the problem that we're trying to address?” (Academic) |
|  | An absence of strategic alignment across partners is detrimental to the multistakeholder process. | “for a partnership to work there has to be a level of alignment, and a level of commitment from both sides to achieving a common outcome. And usually, I mean honestly nine times out of ten, when there isn’t a strategic alignment or an alignment in values and objectives partnerships often don’t succeed” (International Organisation). |
|  | MSIs are not able to recognise and address the fundamental problem, particularly when they lack inclusion of affected communities | “OK, you've had a big impact on the vitamin A indicators in a region. But if that's not what the countries were prioritizing as the real drivers of their […] issue, are you? Is it good enough?” (MSI) |
|  | Focusing on consensual orientation and setting a shared goal among partners might result in less ambitious goals as partners need to compromise. | “If you're going to work across different stakeholder groups, you're going to have to accept less than the gold standard because you have to start somewhere. And when you have groups that start on opposite ends of things, you have to find middle ground and work gradually to be able to get to where you want to go. […] the quote I like is ‘we shouldn't let perfect be the enemy of good’. And so to me that is where it is. How do we find middle ground? How do we find things that are relatively acceptable and palatable to all sides. Not perfect. Maybe not even great, but [that] at least get us moving” (Business-led MSI). |
|  | Even when a partner in the MSI agrees to a shared goal, that does not mean their sector or even organisation is willing to move towards that goal. | “You get an academic on the [multistakeholder] body, […] that doesn't mean that scholar represents even their subspecialty in those conversations. Although that claim is presented as The Academy is backing us” (Academic).  “There's always this dance with one foot in the pragmatic. What's practical? What are you able to do given your job, your role, your boss, your board of directors?” (MSI) |
|  | Conflicts of interest between actors, particularly the private sector, limit the ability of MSIs to have a consensual process | “the question is always do we involve the private sector further because we need them. Or do we take a step back because we know that their interests are just not generally the same as the public general interest? In that their primary motivation is more financial and to be profitable than it is about doing good” (NGO). |
|  | For some organisations, being required to reach consensus with organisations whose mandate and objective might be opposed to their own presents a large reputational risk, which precludes them from engaging in MSIs. | “If [organisation] engages with partners in the food industry, then we in a way are held accountable for their actions as well […] And actually we are disengaging more and more [from] the food industry because of these reputational risks. And there have been multiple studies done in the sense of trying to understand how much an entity like ours can influence the decisions of the food industry. In the engagement that we have with them, how much can we sort of help them be better, you know? And the evidence says that we can’t. We might be able to, a little bit, but the reputational risks and the other risks, they outweigh the potential benefits of these kind of engagements” (International Organisation). |
| **Transparency**  Transparency of structures, processes and results | MSIs are often seen by those external to them as lacking transparency about their results and processes. | “There is no transparency. The transparency is ad hoc. I think I know of only one organisation that publishes its financial statements, who does the minimal. Where is the money coming from? Where is it going to? In particular what are the monetary and non-monetary transactions between major players and everybody else at the table? Things which we would not accept in the public domain.” (Academic) |
|  | Many MSIs do not have the resources to have formalised monitoring processes in place and be transparent about their governance and outcomes. | “We’re not going to be reporting on the impact of [MSI] because that would be probably too ambitious an endeavour for the resources that we have. But more just recording that the [actions] are taking place with the assumption that because the [action] is based on evidence and research that if investments are aligned with that evidence base, then impact will inevitably ensue. […] it was very important that there was accountability built in and we didn't want just random commitments being made that no one was going to track. But we don't really have the resources to be tracking all the commitments. That's not what we do” (NGO) |
|  | MSIs rely on trust relationships rather than more formal monitoring mechanisms | “I guess we trust people. We rely on the wider family telling us when things are going wrong. There are one or two members of [MSI] who we have some doubts and concerns about, so we maybe keep a little bit more of an eye on what they’re doing. […] you know, probably I’m too trusting. But, you know, it hasn’t gone wrong yet.” (MSI) |
|  | There is a lack of trust between partners in the MSI, in part due to a perceived lack of transparency about partners’ motivations for participating in the MSI. | “You’ve got smaller NGOs and then within the core you also have these really big corporations, which, there might historically be a high level of distrust between those organisations. How do you manage that? Again, it’s quite tricky” (MSI)  “Yes there is definitely a conflict of interest with [company]. I actually know the people at [company] quite well and the question is always whether they do it for greenwashing purposes to be part of it and to show to the outside world ‘see we want to go in the right direction.’ And we’re not totally clear yet how we have to view this” (Transnational corporation)  “we have known that people who are interested in moving forward products that compete with health are actually delaying agendas. So if it's so obvious that they are delaying agendas, why would they want to be part of a multistakeholder [initiative] that is supposed to move forward the agenda, you see? So it's a contradiction” (Academic). |
|  | Lines of accountability are unclear in multistakeholder processes, limiting the ability of MSIs to be transparent about their processes and results. | “Accountability becomes very conceptual. But when it comes to the real pragmatic parts of what does that look like? It’s often harder to ascertain” (MSI). |
| **Participants’ broader reflections on multistakeholderism as a global governance approach** | | |
| **Subtheme** | | **Illustrative quotes** |
| Multistakeholderism is perceived as necessary given the widespread corporate capture of public institutions. | | “To me, the issue is that those international organisations that the public believe are representing us are not doing their job in being transparent about the decisions. Who is sitting in those groups, who is represented, you know? […] So to me the failure is from the government. And they are providing too much of a seat or, I don't know, too much power to the industry. With the risk that the industry will capture those organizations” (Academic). |
| Multistakeholderism may have a role in driving global shifts when it is used in conjunction with other, more confrontational approaches. | | “The reason we do that is not because we don’t think protest is effective. We really do. […] our theory of change is that there needs to be some partnership approaches at the same time as there needs to be protest approaches. Because actually, if you looked at it as a whole kind of tapestry of things that are pushing for change, you need those almost conciliatory forces as well as the challenging forces” (MSI). |
| Multistakeholderism has not been able to meet its projected results | | “If MSI's were working, why are we still stuck here, right? They've been developed and these conversations have been happening for a decade, and yet workers are still feeling like things are not moving the direction that they need to. Or their rights are still being violated. And why is that?” (NGO). |
| There is a need to recognise the system constraints that multistakeholder approaches face and that may limit their ability to drive progress. | | “We're trying, and I think there's a difference between ambition and the goal, and the reality. And, you know, you hold the ambition, but you also hold some reality. And so I don't want to seem naive and to think that if you just crack the mould you can now rebuild. Again, there's a lot of incentives, disincentives on both sides, and complexity, to address” (MSI). |
| There is a lack of institutional support in the wider system surrounding multistakeholder initiatives, which significantly limit its ability to function. | | “And this is where I go back to saying I see lots of individuals who are ready for a level of change. Who really want to be part of changing that. But somehow our systemic structures inhibit that. And that's, you know, that's delivering on profit and so on” (MSI). |
| The shift towards multistakeholderism has broader consequences for global governance as it shifts the parameters for governance more generally. | | “slowly there is an erosion of the governance systems already in place. Because those governance systems are now accommodating the private sector and its own definition of conflict of interest, etcetera. And the space for the public is also shrinking in those platforms. And there is little known about, you know, how the decisions are made, etcetera” (Academic). |
| The expectations of multistakeholderism need to be adjusted in light of concerns over the legitimacy of the multistakeholder process. | | “What I think needs to happen with MSIs is to recognise them for what they are. […] Stop suggesting to governments that they are able to do what government was previously expected to do, which is to both regulate and oversee the behaviour of companies. All of that has to go because of the corporate capture of them. But that's not to say they need to go as initiatives themselves. We just need to significantly reimagine and redefine what they're doing” (NGO). |
